# High light-quality OLEDs with a wet-processed single emissive layer

**DOI:** 10.1038/s41598-018-24125-4

**Published:** 2018-05-08

**Authors:** Meenu Singh, Jwo-Huei Jou, Snehasis Sahoo, Sujith S. S., Zhe-Kai He, Gintare Krucaite, Saulius Grigalevicius, Ching-Wu Wang

**Affiliations:** 1 Department of Materials Science and Engineering, National Tsing Hua University, Hsin-Chu, Taiwan Republic of China; 2 Department of Polymer Chemistry and Technology, Kaunas University of Technology, Radvilenu plentas 19, LT50254 Kaunas, Lithuania; 3 Institute of Optoelectronics and Electrical Engineering, National Chung Cheng University, Taiwan, Republic of China

## Abstract

High light-quality and low color temperature are crucial to justify a comfortable healthy illumination. Wet-process enables electronic devices cost-effective fabrication feasibility. We present herein low color temperature, blue-emission hazards free organic light emitting diodes (OLEDs) with very-high light-quality indices, that with a single emissive layer spin-coated with multiple blackbody-radiation complementary dyes, namely deep-red, yellow, green and sky-blue. Specifically, an OLED with a 1,854 K color temperature showed a color rendering index (CRI) of 90 and a spectrum resemblance index (SRI) of 88, whose melatonin suppression sensitivity is only 3% relative to a reference blue light of 480 nm. Its maximum retina permissible exposure limit is 3,454 seconds at 100 lx, 11, 10 and 6 times longer and safer than the counterparts of compact fluorescent lamp (5,920 K), light emitting diode (5,500 K) and OLED (5,000 K). By incorporating a co-host, tris(4-carbazoyl-9-ylphenyl)amine (TCTA), the resulting OLED showed a current efficiency of 24.9 cd/A and an external quantum efficiency of 24.5% at 100 cd/m^2^. It exhibited ultra-high light quality with a CRI of 93 and an SRI of 92. These prove blue-hazard free, high quality and healthy OLED to be fabrication feasible via the easy-to-apply wet-processed single emissive layer with multiple emitters.

## Introduction

Organic light-emitting diodes (OLEDs) have drawn enormous attention due to their increasing applications in flat-panel displays and solid state lightings^1–4^. A wide variety of OLED displays such as mobile phones, tablets, notebooks, TV panels, industrial and professional displays, and micro-display products have already been developed. OLED display has achieved a significant share in the display market and grown to approximate 16-billion dollars in the global sales. It is expected to rise to 57 billions by the year of 2026^5^. OLED lighting application includes various sectors of lighting like residential, outdoor, commercial building, hospitality, and automotive etc. Lately, LG Display has launched warm-white indoor lighting panels of different sizes varying from 53 mm × 55 mm to 320 mm × 320 mm and also a 406 mm × 50 mm flexible panel^6^. Besides, companies such as Kaneka Corporation, Konica Minolta, Acuity brand lighting, MC Pioneer, and OLEDWorks are also manufacturing OLED panels for general purpose illumination. OLED lighting is also growing in global market and the sales is expected to achieve 2.5 billions by the year of 2027^7^.

High-quality light is crucial for illumination in surgery-rooms, photography, museums, art-galleries, and exhibition-halls etc. A high-quality light can easily discriminate the shades of a color and provide the visual comfort by differentiating the objects. In the past, one light quality metric, color rendering index, CRI, had been developed and has been widely adopted for a long while. However, it is found to be inappropriate for describing the quality of light according to U.S. Department of Energy (DOE)^8^. For example, the long applied low-pressure sodium lamp could be given a negative CRI of “−12”^9^. To respond to the call for a new and appropriate light quality metric by DOE, Jou’s group had developed a lighting metric, namely spectrum resemblance index, SRI, which quantitatively defines the quality of any given light from a 0 to 100 scale^10^. To realize a high CRI or SRI, emission in the visible region should be as broad as possible. Since OLED materials can easily generate any desired color with a comparatively diffused and broad spectrum, high-quality light sources can hence be made by using OLED technology with the employment of a few white light complementary emitters.

A number of approaches have been demonstrated in the fabrication of high-quality light OLEDs. For multiple emitters within a single emissive layer, for example, Xie *et al*. reported a polymer OLED with a CRI of 92 and efficacy of 3.0 lm/W at 1,000 cd/m^2^, by incorporating wide-spectrum dopants^11^. For multiple emissive layers, Wu and Chi *et al*.^12^ reported a phosphorescent OLED with a CRI of 94 and efficacy of 3.8 lm/W with three doped emissive layers, and Liu *et al*.^13^ reported a CRI of 91 and efficacy of 3.2 lm/W with three non-doped emissive layers. Moreover, fluorescent and phosphorescent based hybrid systems have been explored to achieve a widespread spectrum^14,15^. For example, Duan *et al*. reported a CRI of 63 and efficacy of 21 lm/W by utilizing a thermally activated delayed florescence host with orange-phosphor in single emissive layer^16^. Lee *et al*. reported a hybrid OLED with a CRI of 77 and external quantum efficiency of 19%^17^. Additionally, a nano carrier modulation layer plays a significant role in achieving a very-high CRI by regulating the carrier injection, widening the recombination zone and controlling the excitons in the desired emissive layers^18–20^. Jou’s group reported a CRI of 96 and efficacy of 5.2 lm/W by employing a carrier modulation layer between the fluorescent and phosphorescent white emissive layers^18^. Later, Jou’s group reported a hybrid OLED with a CRI of 93 and efficacy of 14 lm/W^20^.

The fabrication of OLED device can be done by two distinct methods, namely wet- and dry-process. Typically, dry-process is used to realize high efficacy OLEDs with high-quality light^21,22^. Whilst, shortcomings such as inefficient use of materials, high energy consumption and poor scalability are major issues in cost-effective mass-production. In contrast, some of the aforementioned approaches can be adopted in wet-process to fabricate high-quality light OLED devices, but with comparatively low efficiency. Nevertheless, wet-process is deemed more superior in enabling simple fabrication, large area-size and roll-to-roll production, and consequently more cost-effective.

Not only quality but also color temperature of light has a profound effect on human health, environment and ecosystem. Many medical studies had shown concern about hazards resulting from blue-enriched white light that has a color temperature ranging from 2,500 to 6,500 K. Long exposure to such a white light can easily disrupt human-body clock and result in chronic health issues, such as sleep disorder, obesity, diabetes, breast cancer in women, and prostate cancer in men^23–25^. Moreover, a high color temperature light might damage light-sensitive tissues of eyes and even lead to blindness^26^. The International Dark-Sky Association reported that intensive white light causes light pollution at night and can disrupt the living behavior of nocturnal species^27^. Ironically, most of the modern electric lights exhibit a greater than 2,500 K color temperature. In contrast, very few electricity-driven lighting measures have a color temperature around 2,000 K of candlelight, which has an orange-white appearance and contains little violet or deep-blue emission. Such a low color temperature, blue-hazard free light can be adopted to inhibit those health issues mentioned above. However, it is highly challenging to realize a low color temperature, orange-white emission with a very-high CRI or SRI at the same time since the hazardous violet and deep-blue emission cannot be included.

Currently, a few researchers have been focusing on the development of wet-processable low color temperature, blue-emission hazard free OLEDs with high CRI. Jou’s group developed an OLED with a low color temperature of 2,280 K and a power efficiency of 39 lm/W by spin-coating a white emissive layer^28^. Later, a wet processed OLED with a 1,918 K color temperature was also demonstrated^29^. The device showed a 27 lm/W efficacy with a CRI of 38 at 1000 cd/m^2^. Ye and Liu *et al*. reported a single emissive layer based white OLED with a 3,400 K color temperature and an 82 CRI^30^. Zhang *et al*. reported a 1,880 K OLED with a maximum efficacy of 1.5 lm/W by using a new dye with aggregation-induced emission characteristics^31^. Xie *et al*. reported a novel red phosphorescent dye based white OLED with a 2,331 K color temperature and an 89 CRI^32^. Huang *et al*. reported a newly synthesized red phosphorescent emitter and fabricated a single emission layer white polymer OLED with a 3,400 K color temperature, an 82 CRI and a maximum 9 lm/W efficacy^33^.

In the present work, we demonstrate wet-processed single emissive layer OLEDs which exhibit a low color temperature with very-high CRI and SRI values, show less suppression of melatonin generation and eventually much friendlier to circadian rhythm, provide visual comfort to human eye, and show longer permissible exposure time to retina without causing any damage. To evaluate the impact of lighting on human health, the emitted spectrum of the fabricated OLEDs is characterized by using melatonin suppression sensitivity (MSS) function^34,35^, maximum permissible retina exposure limit^36^ “t” and light-quality, CRI and SRI. We have also studied the effect of employment of a co-host TCTA on the device performance. Additionally, a cross-linkable molecule 3,6-bis(4-vinylphenyl)-9-ethylcarbazole (VPEC)^37^ was incorporated as a hole-transport layer, which facilitated layer-by-layer structure via spin-coating and resulted a healthy illumination with less melatonin-suppression, longer retina exposure time, low color temperature, and very-high light-quality. The present study includes human health consideration to the designing process of light source and provide an easy to apply cost-effective fabrication process for an efficient light source.

## Theoretical

### Maximum permissible exposure limit “t”

It is necessary to limit the duration of retina exposure to a given light source for the protection of eyes against radiation hazards. For that purpose, the International Electrotechnical Commission (IEC) had reported a maximum admissible retina exposure limit “t” (sec)^36^. According to IEC 62471 standard, the exposure limit “t” can be calculated by using the following equation:

1 $$t=\frac{100}{\,{E}_{B}}$$

where, E_B_ is a blue-light weighted irradiance, which is obtained by using blue-light hazard function B(λ) with a spectral irradiance, E_λ_, of a given light source.

The resulting maximum admissible exposure limit “t” with respect to a correlated color temperature of a given light source can be classified into the following four risk groups:

Risk Group 0 {RG0 (“t” > 10,000 s)}

Risk Group 1 {RG1 (10,000 s > “t” > 100 s)}

Risk Group 2 {RG2 (100 s > “t” > 0.25 s)}

Risk Group 3 {RG3 (0.25 > “t”)}

### Melatonin Suppression Sensitivity determination

Jou^34,35^ had presented the first action spectrum for melatonin suppression per quanta (S_PQ_) over the entire visible range. The melatonin suppression power per photon quanta, S_PQ_, for a monochromatic light of wavelength λ can be determined as follows:

2 $${{\rm{S}}}_{{\rm{PQ}}}({\rm{\lambda }})={10}^{({\rm{\lambda }}r-{\rm{\lambda }})/{\rm{C}}}$$

where, λr is the wavelength of a reference light and “C” a constant determined from a fitting curve.

By considering the suppression power from the perspective of visual impression, S_PQ_(λ) is converted into the melatonin suppression power per lux, S_LC_(λ), by using the photopic luminosity function V(λ). The calculation for the action spectrum of melatonin suppression per lux, S_LC_(λ), is given below:

3 $${{\rm{S}}}_{{\rm{LC}}}({\rm{\lambda }})={\rm{\lambda }}\times {{\rm{S}}}_{{\rm{PQ}}}({\rm{\lambda }})/{\rm{V}}({\rm{\lambda }})$$

Relative to the reference light, λr, melatonin suppression sensitivity of a given light can be defined as follows:

4 $${\rm{Melatonin}}\,{\rm{suppression}}\,{\rm{sensitivity}}\,( \% )=\frac{{S}_{LC}({\rm{\lambda }})\,}{\,{S}_{LC}\,({\rm{\lambda }}r)}\times 100$$

### Calculation of the spectrum resemblance index, SRI

Jou *et al*.^10^ reported in 2014 a natural light spectrum resemblance index, SRI, to quantify the quality of a given light source. The obtained power spectrum of a light source is first converted to the luminance spectrum. The resultant spectrum is then directly compared with the blackbody-radiation having the same color temperature. The calculation of SRI can be done as follows:

5 $$\mathrm{SRI}=\frac{\int L(\lambda ,T)\,{\rm{d}}\lambda }{{{\rm{L}}}_{BR}(\lambda ,T)\,{\rm{d}}\lambda }\times 100 \% $$

## Experimental

### Device fabrication

The structure of the OLED device was composed of a glass substrate with a 125 nm layer of indium tin oxide (ITO) anode. An aqueous solution of poly (3,4-ethylenedioxythiophene)-poly(styrenesulfonate) (PEDOT:PSS) was spin-coated on a pre-cleaned ITO anode at 4000 rpm for 20 s to form a hole-injection layer (HIL) of 32 nm. The resulting HIL was dried for 40 mins at 160 °C. The emissive layer (EML) solution consisted of a host 4,4, N N-dicarbazolebiphenyl (CBP) and guest emitters namely deep-red bis(1-phenylisoquinolinolato-C2,N) iridium (acetylacetonate) (Ir(piq)_2_(acac)), yellow Iridium(III) bis(4-phenylthieno[3,2-c]pyridinatoN,C2) acetylacetonate (PO-01), green tris(2phenyl-pyridine) iridium (Ir(ppy)_3_), and sky-blue bis[3,5-difluoro-2-(2-pyridyl)phenyl]-(2carboxypyridyl) iridium(III) (FIrpic). The following EML solution was prepared by dissolving the host and guest materials in tetrahydrofuran solvent and sonicated for 30 mins. The resulting EML solution was spin-coated on the hole-injection layer at 2,500 rpm for 20 s under nitrogen purging condition.

**Figure 1:**
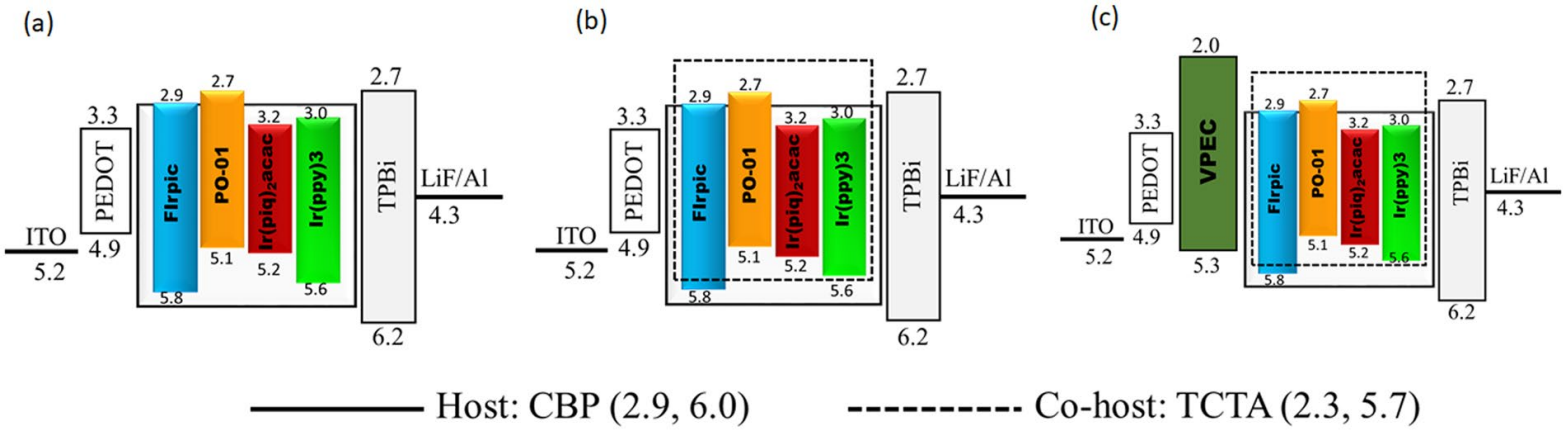
Schematic energy-level diagrams of the studied OLED devices. (**a**) Device with four different white-light complementary emitters, (**b**) devices with the incorporation of a co-host TCTA, and (**c**) devices with a hole-transport layer.

Here, we have fabricated seven OLEDs by following the device architectures as shown in Fig. 1. The EML solution for Device A was composed by 12 wt.% FIrpic, 0.3 wt.% Ir(ppy)_3_, 0.8 wt.% Ir(piq)_2_(acac), and 0.5 wt.% PO-01 doped in the CBP host (Fig. 1(a)). For Device B1, the EML solution was prepared by incorporating a co-host TCTA with CBP host at 3:1 ratio, while the emitter concentrations were kept the same as in Device A, as shown in Fig. 1(b). Further, Devices B2 and B3 were fabricated by varying the doping concentration of the green, deep-red and yellow emitters, while fixing the concentration of FIrpic at 12 wt% in the same host to co-host solution. The resulting EML for Device B2 consisted of 0.7 wt.% Ir(ppy)_3_, 1 wt.% Ir(piq)_2_(acac), and 0.9 wt.% PO-01 and Device B3 consisted of 0.7 wt.% Ir(ppy)3, 1.3 wt.% Ir(piq)_2_(acac), 0.9 wt.% PO-01. Additionally, a hole-transport layer (HTL) of VPEC was incorporated to fabricate Devices C1, C2 and C3 with the same emissive layer composition as in B1, B2 and B3, respectively, as shown in Fig. 1(c). An organic solvent chlorobenzene was used to dissolve VPEC polymer. The resulting solution was sonicated for 30 mins. The prepared solution was spin-coated on the pre-dried HIL layer at 2,500 rpm for 20 s to form a 20 nm HTL. To remove solvent, initially HTL was dried for 20 mins at 120 °C and thereafter heated for 40 mins at 200 °C for crosslinking reaction. Subsequently, a 32 nm electron-transporting layer of 1,3,5-tris(N-phenylbenzimidazol-2-yl)benzene (TPBi), a 1 nm electron injection layer of lithium fluoride, and a 130 nm layer of aluminum as cathode were deposited by using thermal evaporation method in high vacuum chamber (10^−4^ Torr) at respective rates of 0.3, 0.1 and 10 Å/s.

### characterization

All the fabricated devices were measured at room temperature. A Keithley 2400 electrometer with a Minolta CS-100 luminance meter was used to measure the current–voltage (I–V) characteristics. The luminance, CIE chromaticity coordinates, color temperature, and electroluminescence spectrum of the fabricated devices were measured by using a Photo Research PR-655 spectrascan spectroradiometer. The fabricated OLED devices were having an emission area of 25 mm^2^ and the luminance was measured in the forward direction.

## Results and Discussion

**Figure 2:**
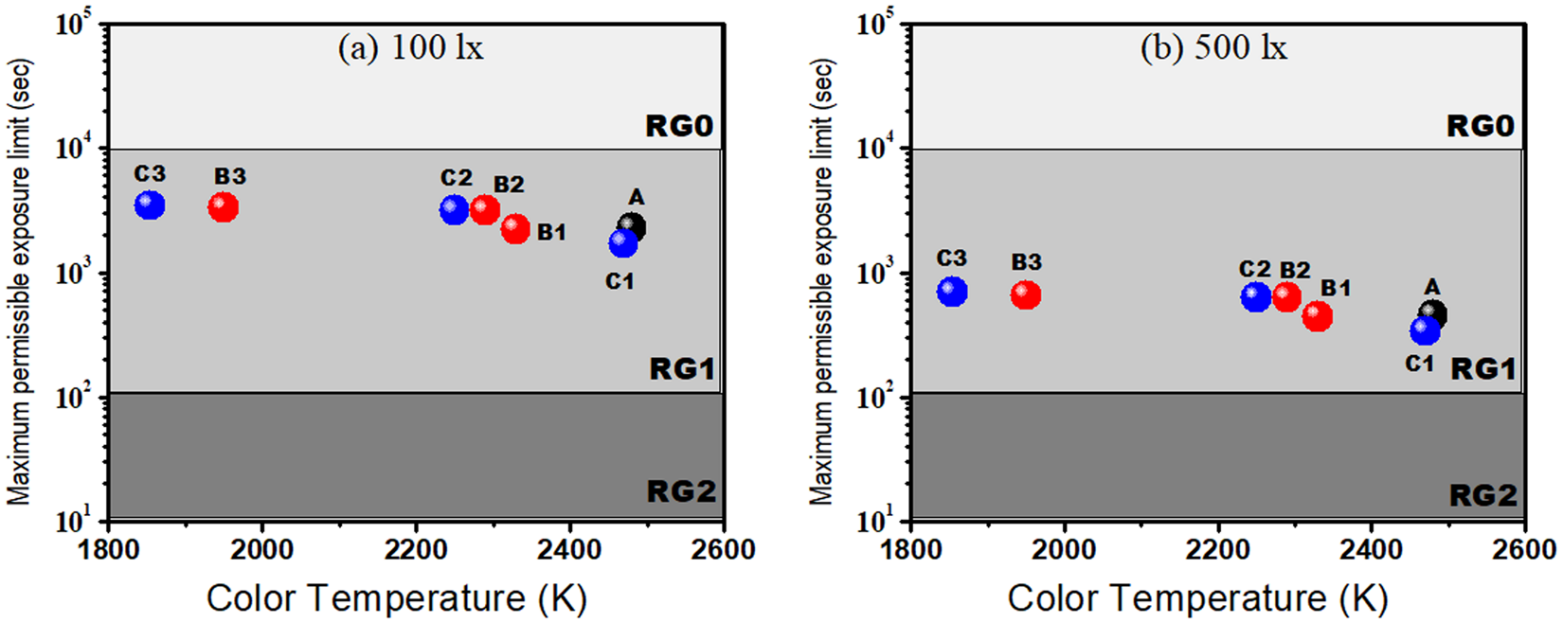
Effect of color temperature of the studied low color temperature OLEDs on the retinal maximum permissible exposure limit at (**a**) 100 and (**b**) 500 lx.

Retinal exposure duration can be affected by color temperature and brightness of a light source, as can be seen in Fig. 2(a and b). Photochemical damage of retina cells can result from a longer exposure to a higher color temperature but less bright light or a shorter exposure to a more intense white light^38^. High color temperature white light consists of short wavelength radiation which can damage retina cells from a minimum 10-seconds exposure to 1–2 hours duration^36^. It should be noted that any device with a low color temperature classified to risk group RG0 is also harmful to the retina, as the exposure time exceeds beyond 10,000 s^36^.

Here, we have examined the emission spectrum of the fabricated OLED devices at 100 lx, a typically used illuminance for household lighting and 500 lx bright light used for commercial purpose. All the fabricated devices showed a risk group of RG1, where exposure limit ranges from 100 s to 10,000 s, as shown in Fig. 2(a). For example, Device A with a 2,480 K color temperature showed a maximum permissible exposure limit of 2,264 s (38 mins). Further, maximum exposures of 2,221 s (37 mins), 3,122 s (52 mins), and 3,262 s (54 mins) were observed from Device B1 (2,330 K), B2 (2,290 K), and B3 (1,950 K), respectively. Similarly, retina cells can tolerate 1,695 s (28 mins), 3,134 s (52 mins), and 3,454 s (57 mins) exposure time from Device C1 (2,470 K), C2 (2,250 K), and C3 (1,854 K), respectively. Device C3 exhibited a low color temperature similar to that of a candle (1,850 K) and showed a longer exposure limit due to the absence of emission in the deep-blue and infrared regions.

Most importantly, the applied illuminance has an extremely profound effect on the retinal maximum permissible exposure limit. At applied illuminance of 500 lx, all the devices showed an exposure limit reduced by 5 times, and the entire exposure limit still falls into the RG1 zone, as shown in Fig. 2(b). For example, Device A (2,480 K) showed a maximum permissible exposure limit of 452 s, which can be assigned to risk group RG1. Generally, the permissible exposure limit would decrease with an increasing emission from deep-blue region, consequently increasing the corresponding color temperature, as shown in Table 1. By comparing the fabricated Device C3 (1,854 K) with the most frequently used cold-white compact fluorescent tube (5, 921 K), a light emitting diode (5,501 K), and an OLED (5,000 K) at 100 lx, the low color temperature OLED showed an 11, 10 and 6 times longer exposure limit than these cold-white counterparts, as can be seen in Table 1.

**Table 1:** Comparison of a fabricated OLED (1,854 K) with existing lighting measures in terms of retina maximum permissible exposure limit “t” (sec) and melatonin suppression sensitivity (%).

Light Source	Maximum permissible exposure limit (s)		Melatonin Suppression Sensitivity (%)
	@ 100 lx	@ 500 lx	
Device C3 (1,854 K)	3454	689	3
Candle (1,850 K)	2616	523	4
Cold-white OLED (5,000 K)	546	109	12
Cold-white LED (5,501 K)	343	67	20
Cold-white CFL (5,921 K)	316	63	29

**Figure 3:**
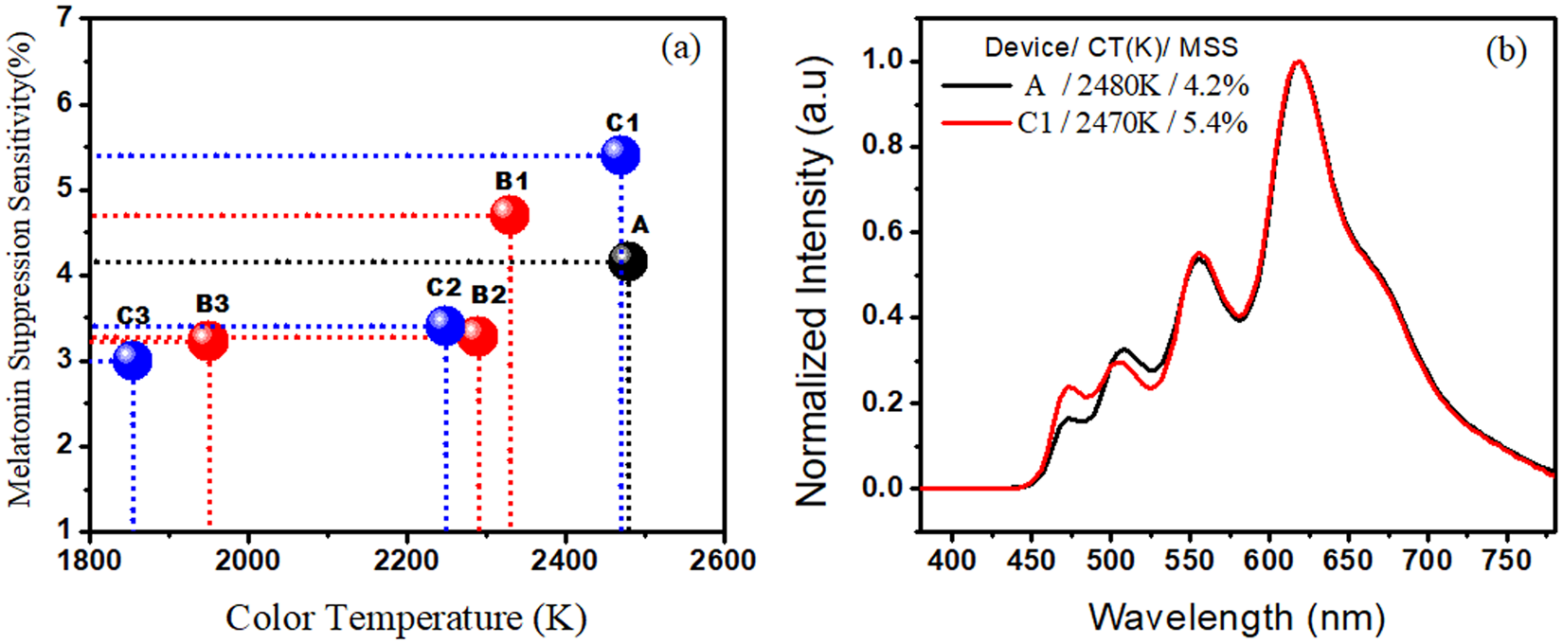
(**a**) Effect of color temperature of the studied wet-processed OLEDs on the melatonin suppression sensitivity (%) and (**b**) comparison of electroluminescence spectra of Device A and Device C1.

As shown in Fig. 3, all the fabricated OLED devices show a melatonin suppression sensitivity below 6%. Device A with a 2,480 K color temperature suppresses the secretion of melatonin to 4.2%. Device B1 (2,330 K), Device B2 (2,290 K) and Device B3 (1,950 K) showed melatonin suppression sensitivity 4.7%, 3.5% and 3.2%, respectively. Further, Device C1 (2,470 K), Device C2 (2,250 K) and Device C3 (1,854 K) suppress the secretion of melatonin to 5.4%, 3.4% and 3%, respectively. Device C3 with a color temperature of 1,854 K suppresses the secretion to 3%, lower than that of a candle (4%), as shown in Table 1. Device C1 (2,470 K) with an almost similar color temperature of Device A (2,480 K) showed a higher suppression sensitivity of 5.4%. The reason why Device C showed a highest suppression sensitivity can be explained by Fig. 3(b). The suppression of melatonin-secretion is not only sensitive to a color temperature but also sensitively dependent on the intensity of short-wavelength radiation^39^. It can also be noted that the suppression power is significantly higher as the emissive wavelength is shorter especially in the deep-blue or violet region. Moreover, by comparing Device C3 (3%) with cold-white CFL (29%), LED (20%) and OLED (12%), it suppresses 90%, 85% and 75% less melatonin-secretion, respectively, as shown in Table 1. The reported low color temperature Device B3 (1,950 K) and C3 (1,854 K), which showed a minimum suppression effect on melatonin-secretion, can be used as human-friendly light-at-night.

**Figure 4:**
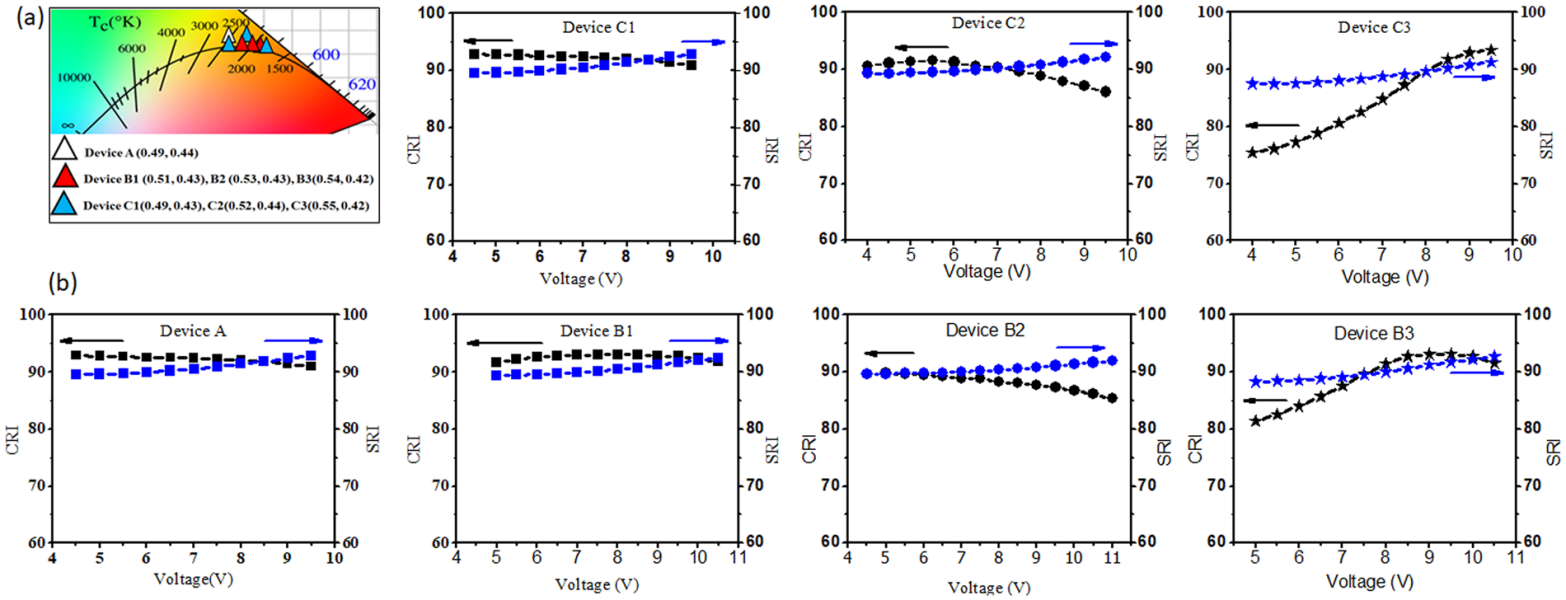
(**a**) Chromaticity coordinates on 1931 CIE diagram and (**b**) color rendering indices versus applied voltage characteristics of the studied wet-processed OLEDs.

Figure 4(a) shows the chromaticity coordinates of the studied low color temperature OLED devices at luminance of 1,000 cd/m^2^. Color coordinates of the resultant devices are analogues to the blackbody radiation and exhibit very-high light quality. All the OLED devices show very-high light-quality indices (CRI and SRI) values throughout the applied voltages, as shown in Fig. 4(b). For example, Device A shows a CRI of 93 and SRI of 90 at 4 V and an 88 CRI and 93 SRI at 9.5 V. Device B1 and Device C1 also show color rendering indices (CRI and SRI) greater than 90 throughout the applied voltages. Device B2 and Device C2 show a slightly varying CRI (90–85) while SRI values remains higher than 90 throughout the applied voltage. For Device B3 and Device C3, there is a significant difference between CRI and SRI values. For example, Device C3 exhibited a very-high SRI value ranges from 87 to 90 at 4 to 10 V, reflecting to be a very-high quality natural light. However, in the same voltage range, CRI varies from 75 to 94. The comparatively lower CRI of 75 can be explained according a prior study^9^, where the calculation of CRI is limited and becomes improper for low color temperature (<1,900 K) testing source.

**Figure 5:**
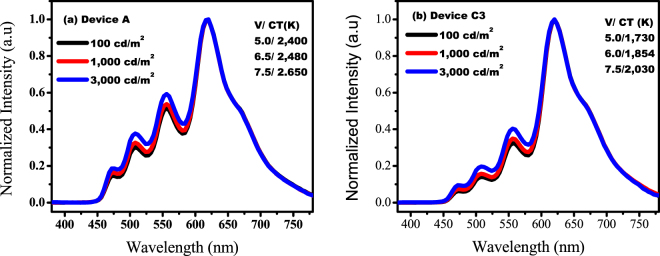
(**a**,**b**) Electroluminescent spectra of the studied wet-processed low color temperature OLED Device A and Device C3 at luminance of 100, 1,000 and 3,000 cd/m^2^.

Figure 5(a and b) show the electroluminescent spectra of the fabricated wet-processed OLED devices A and C3 at luminance of 100, 1,000 and 3,000 cd/m^2^. The resultant devices show extensive emission spectra from 450 to 780 nm. The wide emission spectra may have resulted from the intrinsically broad-band nature of the incorporated organic emitters. The photoluminescent spectra of the four phosphorescent dopants is shown in the electronic supplementary Figure S1(a–b). All the emission spectra show four band emission including a major emission peak in longer wavelength side (beyond 600 nm), which may lead to low color temperature OLEDs, as can be seen in the electronic supplementary Figure S2(a–e). For example, at 100 cd/m^2^, the highest exhibited color temperature is 2,400 K (Device A) and lowest is 1,730 K (Device C3). Furthermore, at 1,000 cd/m^2^, Device A and Device C3 showed a 2,430 K and 1,854 K color temperature, respectively. At 3,000 cd/m^2^, Device A and C3 show a maximum color temperature of 2,650 and 2,030 K, respectively. Device C3 exhibit a significantly low color temperature as compared to incandescent bulbs (2000–2400 K) and even lower color temperature than that of candles (1800–2000 K) at 100 cd/m^2^. It should be noted that all the resultant devices show a small variation of about 200–300 K in color temperature with a varying luminance from 100 to 3,000 cd/m^2^, resulting in stable electroluminescent spectra at varying operation voltage.

**Figure 6:**
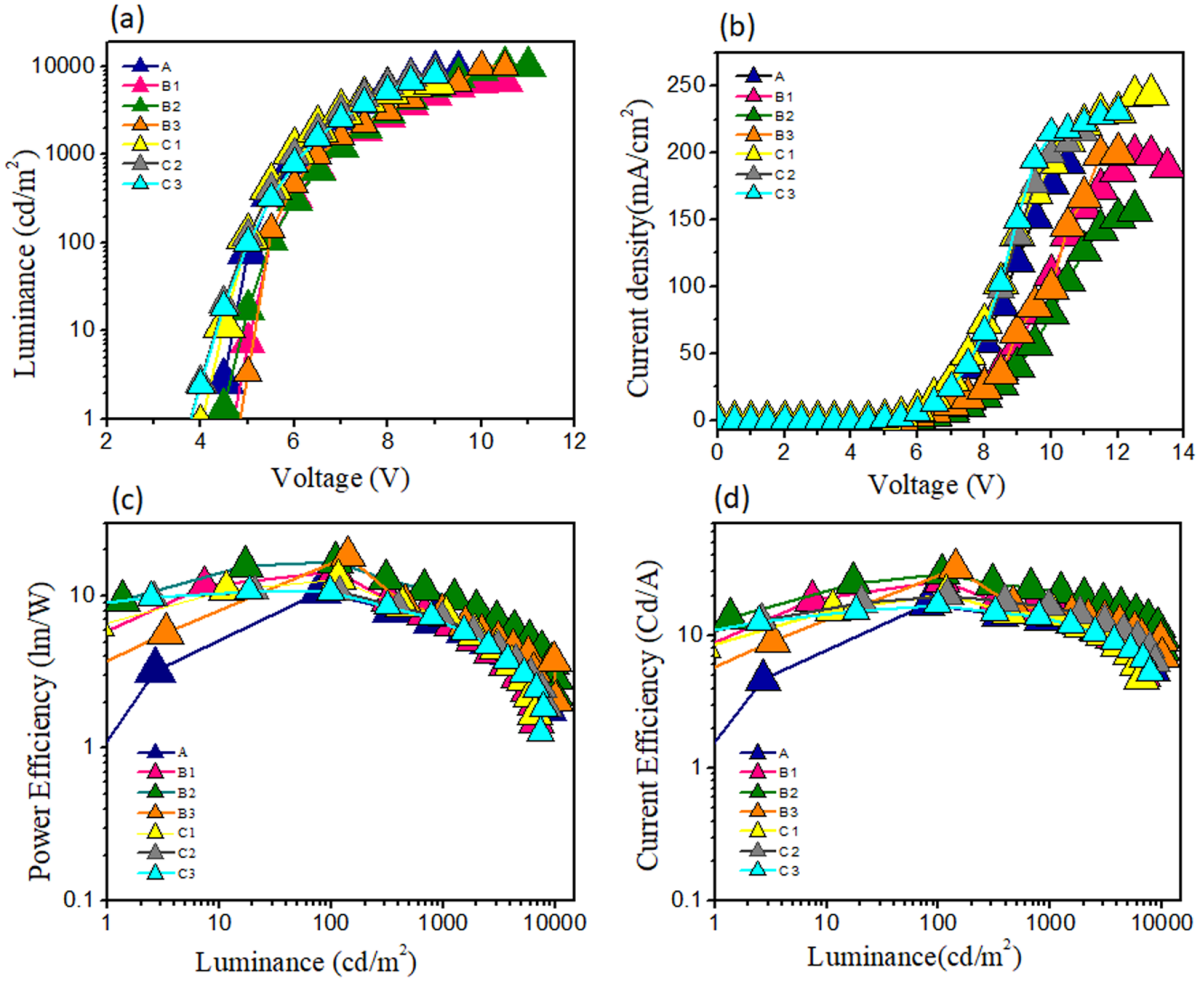
(**a**) Luminance vs. voltage, (**b**) current density vs. voltage, (**c**) power efficiency vs. luminance, and (**d**) current efficiency vs. luminance of the fabricated wet-processed single emissive layer OLED devices.

The current density–voltage–luminance plots of the fabricated OLED devices are shown in Fig. 6(a–d). The corresponding electroluminescent data are given in Table 2. Initially, Device A was fabricated by using 12 wt.% FIrpic, 0.3 wt.% Ir(ppy)_3_, 0.8 wt.% Ir(piq)_2_(acac), and 0.5 wt.% PO-01 doped in the CBP host. Device A exhibited a PE of 9.9 lm/W, a CE of 15.8 cd/A, and an EQE of 11.7% at 5 V and 100 cd/m^2^. The result showed that small band gap dopants: PO-01 and Ir(piq)_2_(acac) enabled direct hole-electron recombination by charge trapping. Additionally, triplet exciton energy-transfer occurs from the emitter Firpic, high triplet energy (2.65 eV), to the CBP host with a comparatively lower triplet energy (2.55 eV), and then from CBP to the other three dyes. The incorporation of high triplet energy (2.74 eV) electron transporting layer of TPBi could enhance radiative recombination by blocking the triplet excitons within the emissive layer. At 1,000 cd/m^2^, Device A exhibited a PE of 5.4 lm/W, a CE of 15.8 cd/A and EQE of 7.3%. It is because hole mobility of CBP (2 × 10^−3^ cm^2 ^V^−1^ s^−1^) is higher than electron mobility of TPBi (3 × 10^−5^ cm^2 ^V^−1^ s^−1^), which could enable charge carrier imbalance at high bias and result in efficiency roll-off at high luminance.

**Table 2:** Effects of co-host, doping concentration and incorporation of a hole transporting layer on the operation voltage (OV), power efficiency (PE), current efficiency (CE), and external quantum efficiency (EQE), CIE coordinates, color temperature (CT), and maximum luminance of the studied wet-procesed OLED devices with a single emissive layer.

Device	Wt.% of dopants				OV (V)	CE (cd/A)	PE (lm/W)	EQE (%)	CIE (x,y)	CT (K)	Max. Lum. (cd/m^2^)
	Firpic	Ir(ppy)^3^	Ir(piq)^2^ acac	PO-01	@ 100/1,000 cd/m^2^						
A	12	0.3	0.8	0.5	5.0/6.1	15.8/10.3	9.9/5.4	11.7/7.3	(0.50, 0.44)/(0.49,0.44)	2400/2480	8312
B1	12	0.3	0.8	0.5	5.5/6.8	24.9/14.7	14.3/6.8	24.5/8.6	(0.52, 0.43)/(0.51,0.43)	2210/2330	6817
B2	12	0.7	1.0	0.9	5.5/6.8	28.6/21.8	16.5/10.1	19.8/12.3	(0.52, 0.44)/(0.53,0.43)	2110/2290	9947
B3	12	0.7	1.3	0.9	5.3/6.5	25/15.6	14.4/7.5	29.4/10.4	(0.54, 0.42)/(0.54,0.42)	1910/1950	9972
C1	12	0.3	0.8	0.5	4.9/5.9	19.6/14.2	12.5/7.5	12.9/8.1	(0.51, 0.42)/(0.49,0.43)	2350/2470	6615
C2	12	0.7	1.0	0.9	4.9/6.0	19.5/16.5	12.5/8.6	13.8/9.5	(0.53, 0.44)/(0.52,0.44)	2160/2250	8674
C3	12	0.7	1.3	0.9	5.0/6.1	16.9/13.4	10.6/6.9	13.4/8.9	(0.56, 0.41)/(0.54,0.42)	1730/1854	7877

For Device B1, a co-host TCTA was incorporated with the host CBP at 3:1 ratio, while the emitter concentrations were kept the same as of Device A. The resultant OLED device showed effective enhancement in efficiencies, for examples, a PE of 14.3 lm/W, a CE of 24.9 cd/A and an EQE of 24.5%. The results indicated that a co-host TCTA not only accelerates the hole and electron trapping for all emitters but also facilitates the energy transfer through host-to guest route for FIrpic. The boosted device efficiency and light emission may be resulted from two mechanisms such as charge carrier trapping in the multiple phosphorescent dopants and langevin recombination^40^. Further, Device B2 is consisted of 0.7 wt.% Ir(ppy)_3_, 1 wt.% Ir(piq)_2_(acac), and 0.9 wt.% PO-01 at fixed concentration of FIrpic (12 wt.%), which shows a PE of 16.5 lm/W, a CE of 28.6 cd/A and EQE of 19.8%. A well optimized concentration of small band gap emitters improved the power and current efficiency, however, slightly decrease the EQE. At 100 cd/m^2^, Device B2 exhibited slightly lower EQE than Device B1, which can be attributed to the increased doping concentration of low band gap emitters resulting into quenching effect at low voltage. However, at high voltage, Device B2 exhibited high efficiencies among all devices. It is because a well optimize doping ratio managed to transfer energy from high band gap to low bandgap emitters, which caused increase in emission intensity in the long wavelength region (beyond 520 nm), as shown in electronic supplementary Figure S2 (b). The resulting OLED B2 exhibited high efficiencies than Device B1 and showed lower efficiency roll-off with increasing luminance, as shown in the electronic supplementary Figure S3(a). Device B3 is consisted of 0.7 wt.% Ir(ppy)_3_, 1.3 wt.% Ir(piq)_2_(acac) at fixed concentration of PO-01 (0.9 wt.%) and FIrpic (12 wt.%). It exhibits a PE of 14.5 lm/W, a CE of 25 cd/A and an EQE of 29.4% at 100 cd/m^2^, making which a very efficient single emissive layer based low color temperature wet-processed OLED^31–33^. It should be noted that all the co-host TCTA based devices showed around 40% decrease in current efficiency and 60–65% drop in EQE at 1,000 cd/m^2^. Hole mobility of TCTA (3 × 10^−3^ cm^2 ^V^−1^ s^−1^) is higher than electron mobility of TPBi, which can boost the accumulation of more holes in the emissive layer at high bias voltage. Therefore, at high luminance, efficiency roll-off in all the reported Devices may be due to field-induced quenching and exciton-polaron quenching^41–43^. A sharp fall in EQE with increasing current density could be due to electrical loss and triplet-triplet annihilation^43,44^, as shown in the electronic supplementary Figures S4(a–b).

Additionally, Devices C1, C2 and C3 were fabricated by incorporating a hole-transporting layer of VPEC with the same emissive layer composition as in B1, B2 and B3, respectively. Devices C1 and C2 exhibited an EQE of 12.9 and 13.8% at 100 cd/m^2^, respectively. Both devices showed a CE of 19 cd/A and a PE of 12.5 lm/W. Further, Device C3 showed a PE of 10.6 lm/W, a CE of 16.9 cd/A and an EQE of 13.4%. Devices C1, C2 and C3 exhibited a slightly lower operational voltage as comparing to Devices B1, B2 and B3. The resultant comparatively low driving voltage may be due to the shallow HOMO level (−5.3 eV) of VPEC, which is 0.7 and 0.4 eV more shallow than that of CBP host and TCTA co-host, respectively. It should be noted that Devices C1, C2 and C3 showed only a 20–25% decrease in CE and a 30–35% decrease in EQE from 100 to 1,000 cd/m^2^.

## Conclusion

In conclusion, this study demonstrates seven low color temperature wet-processed OLEDs with a longer permissible exposure time to human eyes and less suppression effect on the melatonin-secretion as well as a stable and ultra-high light quality emission. A low color temperature OLED (1,854 K) showed a CRI of 90 and an SRI of 88, with a melatonin suppression sensitivity of 3% relative to a reference blue light of 480 nm, which is even lower than that of candles. Its maximum retina permissible exposure limit is 3,454 seconds at 100 lx, which is 11, 10 and 6 times longer and hence safer than the cold-white compact fluorescent lamp (5,920 K), light emitting diode (5,500 K) and OLED (5,000 K). With the incorporation of a co-host and appropriate doping concentration of phosphorescent dopants, an EQE of 20 to 29% with a color temperature ranging from 2,330 to 1,910 K can be obtained. A single emissive layer with four blackbody-radiation complementary dopants provides broad emission range and a stable spectrum with a very-high (>90) CRI and SRI values. These low color temperature, very-high light quality OLEDs may enable future innovations in research and development for human- and environment-friendly lighting sources via such an easy-to-apply and cost-effective wet-processing method.

## Electronic supplementary material

Supplementary Information
