# Supplementary material for: High light-quality OLEDs with a wet-processed single emissive layer
Source: Sci Rep. 2018 May 8;8:7133. doi: 10.1038/s41598-018-24125-4 (PMC5940822; doi:10.1038/s41598-018-24125-4)
Supplement: Supplementary file 1 — Supplementary Information [file 41598_2018_24125_MOESM1_ESM.pdf]

Supporting information

High light-quality OLEDs with a wet-processed single emissive layer

Meenu Singh,[1] Jwo-Huei Jou,[*1] Snehasis Sahoo,[1] Sujith S.S.,[1] Zhe-Kai He,[1] Gintare Krucaite,[2] Saulius Grigalevicius[2], Ching-Wu Wang[3]

[1]Department of Materials Science and Engineering, National Tsing Hua University, Hsin-Chu, Taiwan, Republic of China

[2]Department of Polymer Chemistry and Technology, Kaunas University of Technology, Radvilenu plentas 19, LT50254 Kaunas, Lithuania

[3]Institute of Optoelectronics and Electrical Engineering, National Chung Cheng University Taiwan, Republic of China

*E-mail: jjou@mx.nthu.edu.tw

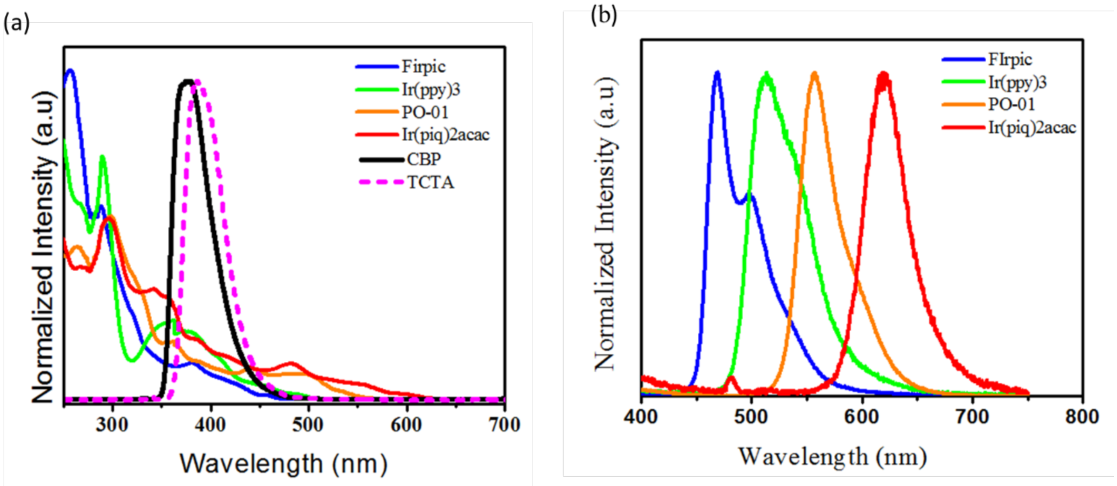

Fig. S1. Photophysical properties of the sky-blue, green, yellow, and deep-red phosphors in tetrahydrofuran solvent: UV-Vis absorption spectra (a) and PL spectra (b).

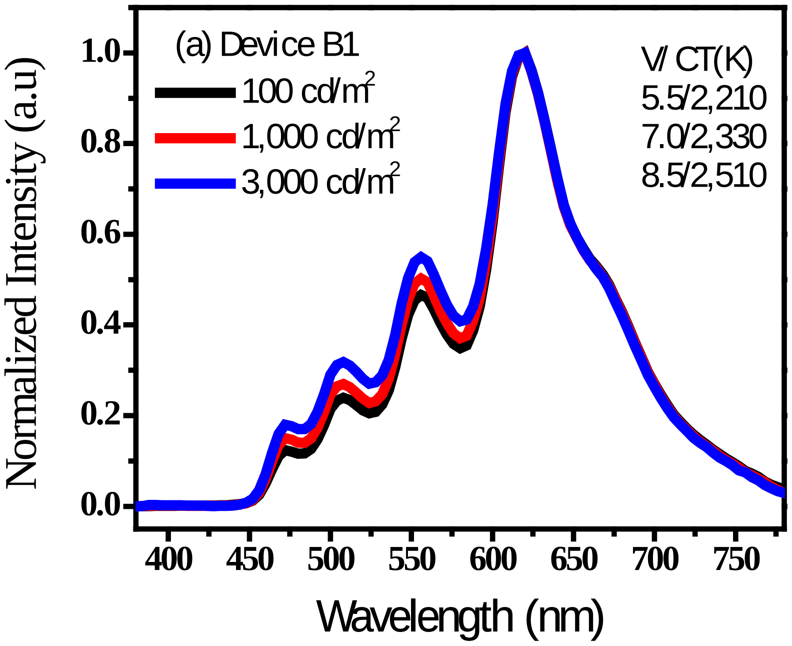
(a) Device B1
100 cd/m2
1,000 cd/m2
3,000 cd/m2
V/ CT(K)
5.5/2,210
7.0/2,330
8.5/2,510
Normalized Intensity (a.u)
Wavelength (nm)
0.0
0.2
0.4
0.6
0.8
1.0
400
450
500
550
600
650
700
750

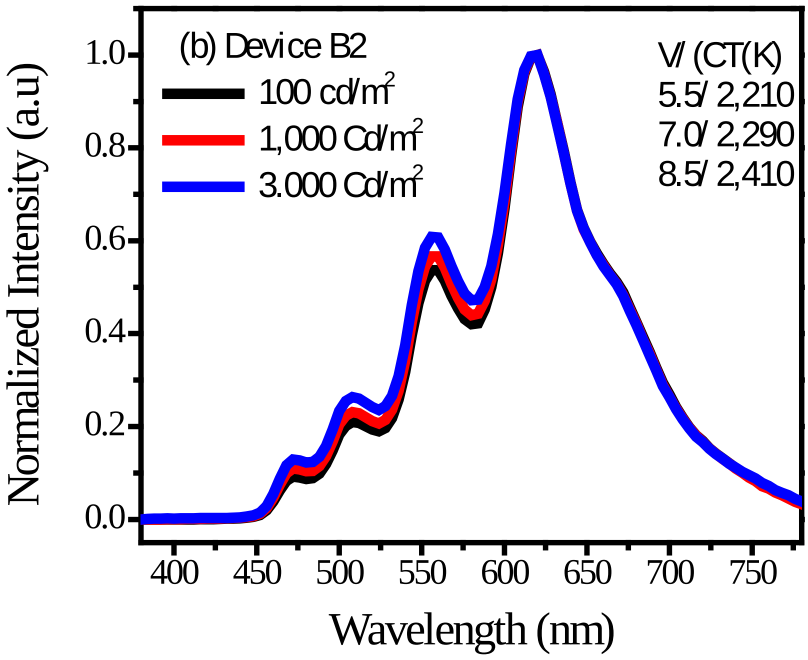
(b) Device B2
100 cd/m2
1,000 Cd/m2
3.000 Cd/m2
V/ (CT(K)
5.5/ 2,210
7.0/ 2,290
8.5/ 2,410
Normalized Intensity (a.u)
Wavelength (nm)
0.0
0.2
0.4
0.6
0.8
1.0
400
450
500
550
600
650
700
750

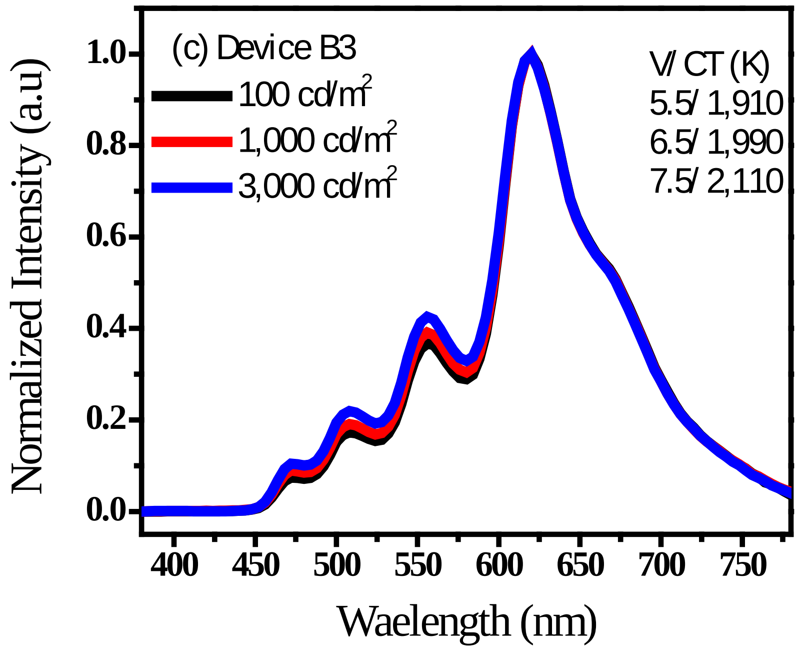
(c) Device B3
100 cd/m2
1,000 cd/m2
3,000 cd/m2
V/ CT (K)
5.5/ 1,910
6.5/ 1,990
7.5/ 2,110
Normalized Intensity (a.u)
Waelength (nm)
1.0
0.8
0.6
0.4
0.2
0.0
400
450
500
550
600
650
700
750

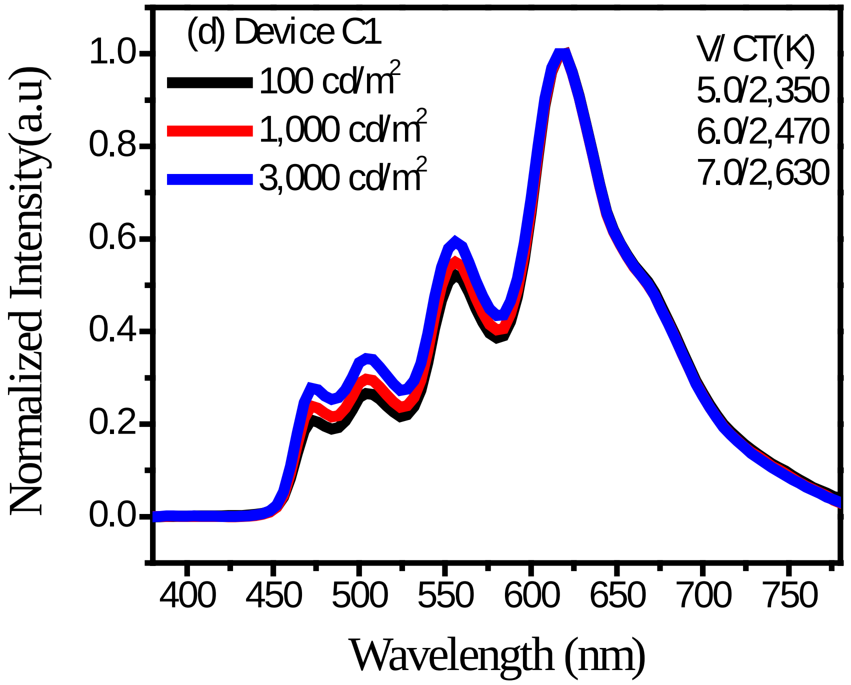
(d) Device C1
100 cd/m2
1,000 cd/m2
3,000 cd/m2
V/ CT(K)
5.0/2,350
6.0/2,470
7.0/2,630
Normalized Intensity(a.u)
Wavelength (nm)
1.0
0.8
0.6
0.4
0.2
0.0
400
450
500
550
600
650
700
750

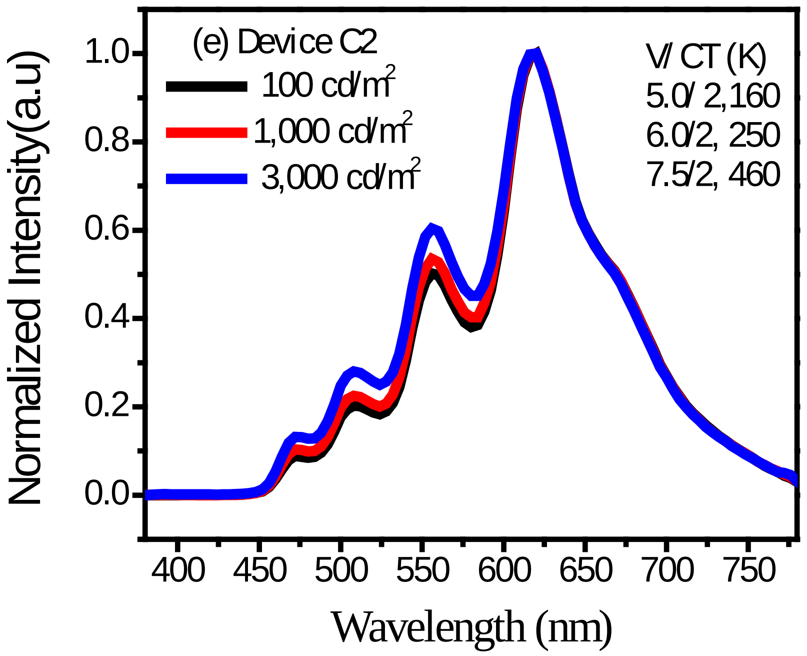

Fig.S2 (a-e). Electroluminescent spectra of the studied wet-processed low color temperature OLED devices at luminance of 100, 1,000 and 3,000 cd/m^2.

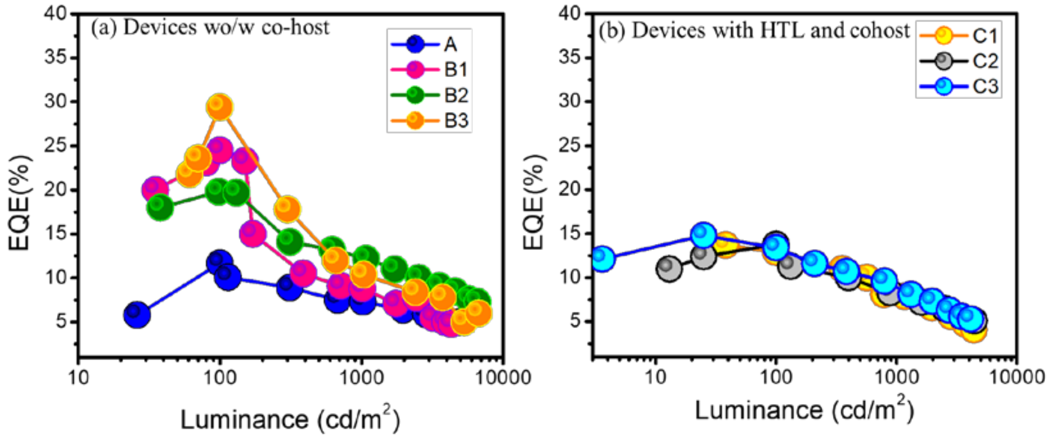

Fig.S3 (a-b). EQE vs. luminance plots of the studied wet-processed low color temperature OLED devices.

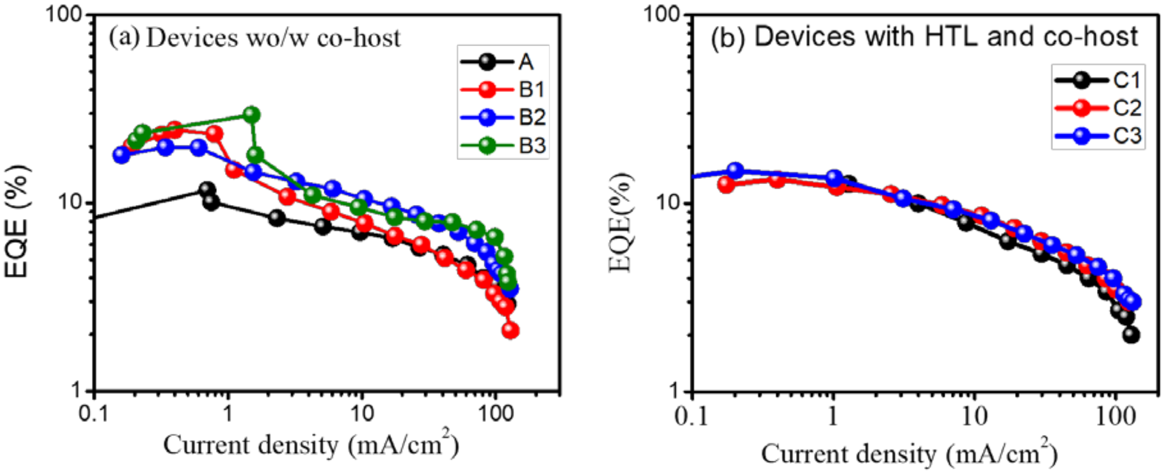

Fig.S4 (a-b). EQE vs. current density plots of the studied wet-processed low color temperature OLED devices.
